# First report of *Echinococcus multilocularis* in cats in Poland: a monitoring study in cats and dogs from a rural area and animal shelter in a highly endemic region

**DOI:** 10.1186/s13071-019-3573-x

**Published:** 2019-06-24

**Authors:** Jacek Karamon, Jacek Sroka, Joanna Dąbrowska, Ewa Bilska-Zając, Jolanta Zdybel, Maciej Kochanowski, Mirosław Różycki, Tomasz Cencek

**Affiliations:** National Veterinary Research Institute, al. Partyzantów 57, 24-100 Pulawy, Poland

**Keywords:** Cat, Dog, *Echinococcus multilocularis*, Helminths, Hookworms, *Mesocestoides*, *Taenia*, *Toxocara*, Poland

## Abstract

**Background:**

Alveolar echinococcosis is a dangerous zoonotic disease caused by larval forms of *Echinococcus multilocularis*. In its life-cycle, the principal definitive host is the red fox; however, domesticated carnivorous animals (dogs and cats) can also act as definitive hosts. Until now, there were no data concerning this infection in cats in Poland. The aim of this study was to estimate the prevalence of *E. multilocularis* in cats and dogs originating from rural areas and animal shelters in a region characterised by a high prevalence of *E. multilocularis* in red foxes.

**Methods:**

Samples of faeces were collected from 67 cats and 268 dogs from a rural area (villages and animal shelters) of a highly endemic region in southeastern Poland. Samples were examined using nested PCR (*E. multilocularis*), multiplex PCR (*E. multilocularis*, *Taenia* spp.) and PCR [*E. granulosus* (*s.l.*)]. Additionally, faeces were examined microscopically (flotation). Moreover, intestines from 110 red foxes shot in the investigated area were examined (sedimentation and counting technique).

**Results:**

Positive PCR results for *E. multilocularis* were obtained in 4 cats (6.0%) and 4 dogs (1.5%). There were no significant differences between groups of animals (from a shelter and with an owner) concerning the prevalence of *E. multilocularis* in both cats and dogs. *Taenia* spp. were found in 10 cats (14.9%) (*Taenia taeniaeformis* and *T. hydatigena*) and 26 dogs (9.7%) (*T. hydatigena*, *T. serialis*, *T. taeniaeformis*, *T. crassiceps*, *T. pisiformis* and *T. ovis*) and *Mesocestoides litteratus* was found in 4 cats (6.0%) and 3 dogs (1.1%). All samples were negative for *E. granulosus* by PCR. Taking into consideration PCR and flotation results, 29 cats (43.3%) and 73 dogs (27.2%) were infected with helminths (26.9 and 11.9%, respectively, were infected with tapeworms). The highly endemic status of the investigated area was confirmed by examination of red foxes: 48.2% of examined red foxes were infected with *E. multilocularis*.

**Conclusions:**

To the best of our knowledge, this study reports the presence of *E. multilocularis* in cats for the first time in Poland and confirms the role of dogs in this infection in highly endemic areas.

## Background

Alveolar echinococcosis (AE) is a dangerous zoonotic disease caused by larval forms of *Echinococcus multilocularis* that develop mostly in the liver. In its life-cycle humans are accidental intermediate hosts; typically, this role is played by rodents. However, the principal definitive host is the red fox, in which adult worms produce eggs in the intestines that are shed with faeces into the environment. Invasive eggs dispersed by the definitive hosts are the source of infection for intermediate hosts and for people. In addition to red foxes, there are other species that are definitive hosts of *E. multilocularis* in wildlife (raccoon dogs, wolves, jackals, arctic foxes) and among domesticated carnivorous animals (dogs and cats). Dogs are characterised by a susceptibility to parasite infection similar to that of foxes: they have similar period of excreting eggs [1]. However, the prevalence among dogs in endemic areas is usually several times lower than that in foxes [2–5], most likely because their eating habits do not expose them in the same way to the larval forms contained within the intermediate hosts. In cats, the infection in the intestines after ingestion of infected rodents (larvae) develops in a limited extent. Although the prepatent period is shorter and egg production is lower, cats could be an important source of infection for humans [1, 6]. In Europe, there have been several reports of *E. multilocularis* detected in dogs from highly endemic regions [2, 7–10]. Furthermore, cases of this infection in cats have also been reported [3, 5, 11–14], which indicates that these animals cannot be ignored as potential sources of infection for people.

Poland is characterised by a relatively high *E. multilocularis* prevalence in red foxes (16%) [15, 16] with several highly endemic regions in the eastern part of the country (up to 48%). Studies conducted a few years ago in southwestern Poland in the area of four districts (powiats) showed *E. multilocularis* infection in dogs for the first time [10]. However, until now, there were no data concerning this infection in cats in Poland. The only study in Poland (based on necropsy of 40 cats) was carried out approximately 20 years ago [17] and had negative results.

The aim of this study was to estimate the prevalence of *E. multilocularis* in cats and dogs originating from rural areas and animal shelter in a region characterised by a high prevalence of this tapeworm in red foxes.

## Methods

### Samples

#### Cats and dogs

**Fig. 1:**
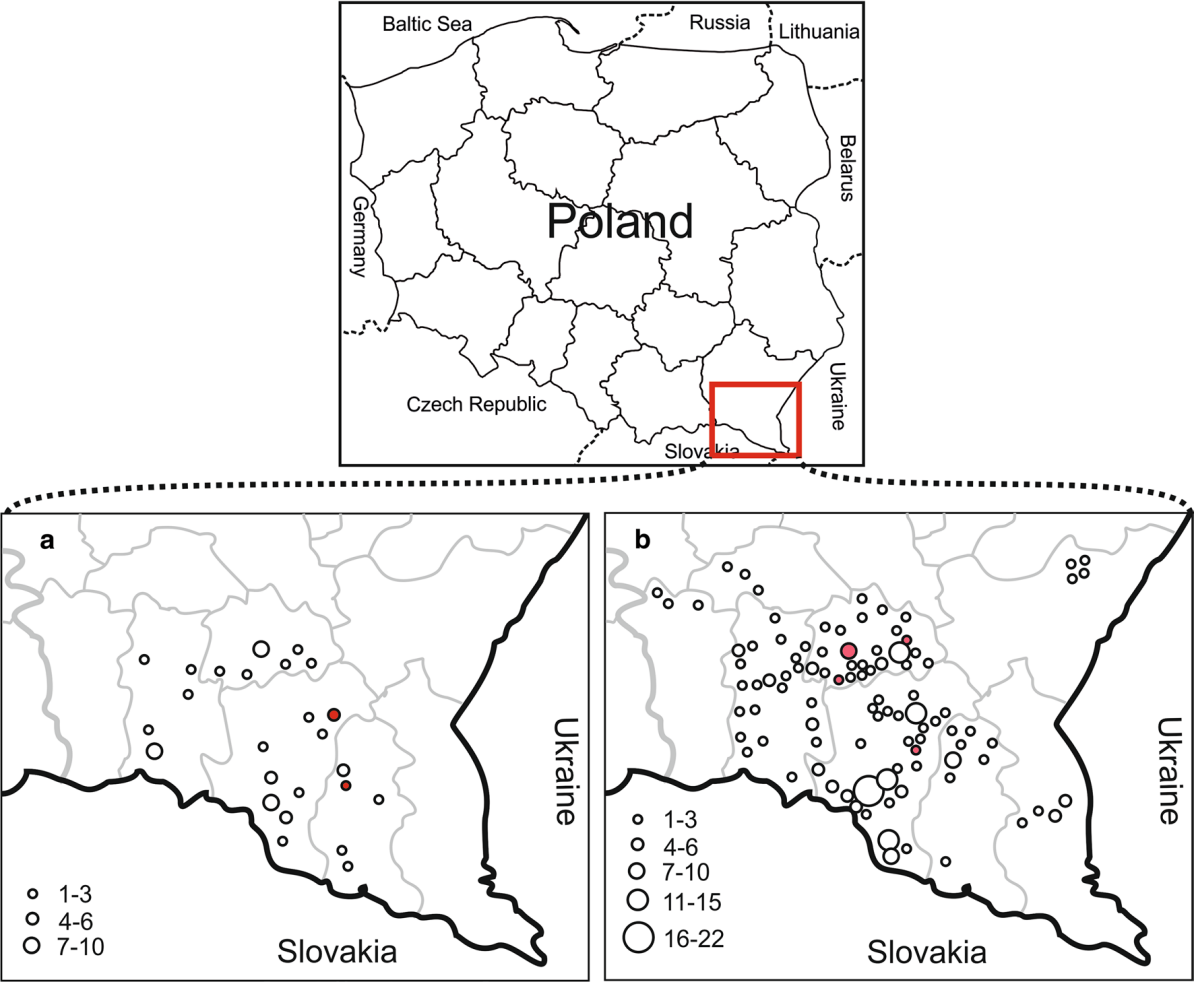
Distribution of the location of collected cats (**a**) and dogs (**b**) faecal samples. Numbers of animals obtained in individual locations are schematically presented by circles of different sizes. Red circles indicate locations where cats/dogs positive for *E. multilocularis* were detected

Samples of faeces were collected from 67 cats and 268 dogs originating from the area of southern part of Podkarpackie Province (NUTS3 PL323) (southeastern Poland) (Fig. 1), between March 2017 and June 2018. Samples were obtained from animals in villages, farms and rural areas of small towns (39 cats and 145 dogs). These faeces were collected by veterinarians during their visits to individual locations. Moreover, samples were collected in quarantine cages of an animal shelter located in Lesko town (Podkarpackie Province, Lesko Disctrict) from newly caught animals (28 cats and 123 dogs) (before deworming) originating from the investigated area described above. Data were collected concerning the location, age and sex of the animals and additionally (in the case of privately-owned animals) antiparasitic treatment. All specimens were frozen at − 20 °C and transferred to the laboratory up to 48 h after sampling.

#### Red foxes

To confirm the high prevalence of *E. multilocularis* in red foxes previously observed in this region [15], small intestines from 110 red foxes shot in the investigated area (NUTS3 PL323) from October 2016 to February 2017 were collected.

### PCR

In the laboratory, faeces were additionally frozen for at least seven days at − 80 °C before examination for safety reasons. DNA from faecal samples was extracted with a QIAamp^®^ DNA Stool Mini Kit (Qiagen, Hilden, Germany) according to the manufacturer’s protocol for larger volumes of stool. First, 10 ml of InhibitEX Buffer (Qiagen) was added to 1 g of sample and thoroughly homogenised (by vortexing with glass beads for 5 min). Next, 2 ml of mixture was used in the following extraction stages. Notably, the temperature of the first incubation was 95 °C, since this was the option recommended in the protocol for samples difficult to lyse. DNA isolates were examined by multiplex PCR with the use of primers to obtain specific products for *E. multilocularis* and *Taenia* spp. [18]. To detect *E. granulosus* (*s.l.*) each sample was examined by PCR according to [19] using only one pair of primers: Eg1121a and Eg1122a. Additionally, samples were tested by nested PCR [20] with some modifications [21] to identify *E. multilocularis*. Internal control (DNA extracted from *E. multilocularis* adult worms isolated from fox intestine) was used to check the inhibition of PCR. For that reason, each DNA sample was examined in duplicate (internal controls were added to duplicate). If a specific band was observed, it confirmed that there was no inhibition.

The positive PCR products were sequenced. Samples for sequencing were purified using Sephadex G-50 columns. Sequencing was performed using a BigDyeTM Terminator v.3.1 Cycle Sequencing kit (Applied Biosystems, Foster City, CA, USA) on an ABI3730xl Genetic Analyzer (Applied Biosystems). The sequenced data were analysed and compared to the GenBank collection using BLAST searches.

### Coproscopy

A portion of each faecal sample (1–2 g) was examined by flotation applying McMaster method according to Raynaud’s modification [22] with the use of saturated natrium chloride solution supplemented with sugar (1 l of saturated NaCl + 500 g of sugar, specific gravity 1.3) to detect parasite eggs.

### Sedimentation and counting technique (SCT)

The small intestines of red foxes were investigated after freezing for at least 10 days at − 80 °C before examination for safety reasons. All intestines were examined using SCT [23, 24].

### Statistical analysis

Differences in the prevalence of the individual infections among shelter and privately-owned animals were assessed by a Chi-square test (or Chi-square with Yates correction). Moreover, the differences in helminths prevalence by sex (male and female), age (> 1 year and ≤ 1 year) and deworming status (dewormed and not dewormed) were tested by a Chi-square test (or Chi-square with Yates correction). Confidence intervals of the percentages of infected foxes were calculated according to the method described by Newcombe [25]. The distribution of quantitative variables was tested by the Shapiro–Wilk test and the normality hypothesis of the data was rejected. The differences in all analyses were considered statistically significant when *P* < 0.05. Statistical analyses were performed using Statistica v.9.1 (StatSoft Inc., Tulsa, OK, USA).

## Results

The results of the molecular investigation are presented in Tables 1 and 2. DNA of *E. multilocularis* was detected in four cats (6.0%), all from shelter cages. *Echinococcus multilocularis*-positive PCR results were also observed in four dogs (1.5%) but in a similar proportion to that of privately-owned and shelter dogs: 1.4% (2 dogs) and 1.6% (2 dogs), respectively. Among all eight positive *E. multilocularis* samples, the specific PCR product in three (2 cats and 1 dog) animals was obtained only with nested PCR; in three other animals (2 cats and 1 dog), the specific product was obtained with both nested PCR and multiplex PCR; and in 2 samples from dogs the product was obtained only with multiplex PCR. The amplicons were sequenced and then compared to those in the GenBank database, confirming that they were *E. multilocularis*. Internal controls showed no inhibition of PCR reaction in any sample.

**Table 1:** Results of molecular analysis (positive samples from PCR and sequencing) and coproscopic examination of cat faeces

Technique	Parasite	Privately-owned cats (*N* = 39)		Shelter cats (*N* = 28)		Total (*N* = 67)	
		*n*	% (95% CI)	*n*	% (95% CI)	*n*	% (95% CI)
PCR	*Echinococcus multilocularis*	0	0.0 (0.0–0.1)	4	14.3 (5.7–31.5)	4	6.0 (2.4–14.4)
	*Taenia* spp.^a^	8	20.5 (10.8–35.5)	2	7.1 (2.0–22.6)	10	14.9 (8.3–25.3)
	*Mesocestoides litteratus*	2	5.1 (1.4–16.9)	2	7.1 (2.0–22.6)	4	6.0 (2.4–14.4)
Coproscopy	Taeniidae	6	15.4 (7.2–29.7)	0	0.0 (0.0–12.1)	6	9.0 (4.2–18.2)
	*Capillaria*	3	7.7 (2.7–20.3)	1	3.6 (0.1–17.7)	4	6.0 (2.4–14.4)
	*Toxocara cati*	8	20.5 (10.8–35.5)	10	35.3 (20.7–54.2)	18	26.9 (17.7–38.5)
	Total nematodes	10	25.6 (14.6–46.4)	11	39.3 (23.6–57.6)	21	31.3 (21.5–43.2)
	Total helminths	12	30.8 (18.6–46.4)	11	39.3 (23.6–57.6)	23	34.3 (24.1–46.3)
PCR and coproscopy	Total tapeworms	11	28.2 (16.6–43.8)	7	25.0 (12.7–43.4)	18	26.9 (17.7–38.5)
	Total helminths	16	41.0 (27.1–56.6)	13	46.4 (29.5–64.2)	29	43.3 (32.1–55.2)

^a^*Taenia taeniaeformis* (7 privately-owned cats and 2 shelter cats), *T. hydatigena* (one privately-owned cat)

**Table 2:** Results of molecular analysis (positive samples from PCR and sequencing) and coproscopic examination of dog faeces

Technique	Parasite	Privately owned dogs (*N* = 145)		Shelter dogs (*N* = 123)		Total (*N* = 268)	
		*n*	% (95% CI)	*n*	% (95% CI)	*n*	% (95% CI)
PCR	*Echinococcus multilocularis*	2	1.4 (0.4–4.9)	2	1.6 (0.5–5.7)	4	1.5 (0.6–3.8)
	*Taenia* spp.^a^	15	10.3 (6.4–16.4)	11	8.9 (5.1–15.3)	26	9.7 (6.7–13.8)
	*Mesocestoides litteratus*	1	0.7 (0.1–3.8)	2	1.6 (0.5–5.7)	3	1.1 (0.4–3.2)
Coproscopy	Taeniidae	3	2.1 (0.7–5.9)	6	4.9 (2.3–10.2)	9	3.4 (1.8–6.3)
	*Capillaria/Trichuris*	13	9.0 (5.3–14.7)	16	13.0 (8.2–20.1)	29	10.8 (7.6–15.1)
	*Toxocara canis*	7	4.8 (2.4–9.6)	13	10.6 (6.3–17.3)	20	7.5 (4.9–11.2)
	*Toxascaris leonina*	1	0.7 (0.1–3.8)	0	0.0 (0.0–3.0)	1	0.4 (0.1–2.1)
	Hookworms	0	0.0 (0.0–2.6)	2	1.6 (0.5–5.7)	2	0.7 (0.2–2.7)
	Total nematodes	18	12.4 (8.0–18.7)^B^	29	23.6 (17.0–31.8)^b^	47	17.5 (13.5–22.5)
	Total helminths	19	13.1 (8.6–19.6)^C^	35	28.5 (21.2–36.9)^c^	54	20.1 (15.8–25.4)
PCR and coproscopy	Tptal tapeworms	18	12.4 (8.0–18.7)	14	11.4 (6.9–18.2)	32	11.9 (8.6–16.4)
	Total helminths	33	22.8 (16.7–30.2)	40	32.5 (24.9–41.2)	73	27.2 (22.3–32.9)

^a^*Taenia hydatigena* (11 privately-owned dogs and 3 shelter dogs), *T. serialis* (5 shelter dogs), *T. taeniaeformis* (2 privately-owned dogs and 1 shelter dog), *T. crassiceps* (2 privately-owned dogs), *T. pisiformis* (1 shelter dog), *T. ovis* (1 shelter dog)

^b, B^ *χ*^2^ = 9.75, *df* =1, *P* = 0.0018 (statistically significant difference between privately-owned and shelter dogs)

^c, C^ *χ*^2^ = 5.73, *df* =1, *P* = 0.0166 (statistically significant difference between privately-owned and shelter dogs)

A *Taenia* spp.-specific product in multiplex PCR (267 bp) was found in 14 cats and 29 dogs. A comparison of sequences with the GenBank database identified nine cats (13.4%) as having *T. taeniaeformis* and one as having *T. hydatigena* (1.5%). In one of the cats, *E. multilocularis* and *T. taeniaeformis* were detected together. In dogs the following *Taenia* species were identified: *T. hydatigena* (14 dogs, 5.2%), *T. serialis* (5 dogs, 1.8%), *T. taeniaeformis* (3 dogs, 1.1%), *T. crassiceps* (2 dogs, 0.7%), *T. pisiformis* (1 dog, 0.4 %) and *T. ovis* (1 dog, 0.4 %). Moreover, *Mesocestoides litteratus* was identified in 3 samples from dogs (1.1%). In one of the dogs, *E. multilocularis* and *M. litteratus* were detected together. None of dogs and cats were positive for *E. granulosus* (*s.l.*) by PCR.

In total, helminth eggs were detected in 32.3% of cats. Tapeworm eggs of taeniid species (including the genera *Taenia* and *Echinococcus*) were found in 9.0% of cats (only in privately-owned cats); none of them were positive for *E. multilocularis* in PCR. Moreover, eggs of *Toxocara cati* (26.9%) and *Capillaria* spp. (6.0%) were also found. A combination of PCR and flotation results showed that helminths analysed together were detected in 43.3% of cats (and tapeworms in 26.0%) (Table 1).

In dogs, helminth eggs were detected in 20.1% of samples. Taeniid eggs were found in 3.4% of dogs. Two dogs shedding taeniid eggs were also positive for *E. multilocularis* by PCR (and negative for *Taenia* spp.). Other parasite species found in dogs by coproscopy were *Capillaria/Trichuris* (these two genera were analysed together because of similar egg morphology) (10.8%), *Toxocara canis* (7.5%), hookworms (0.7%) and *Toxascaris leonina* (0.4%).

The statistical comparisons between groups of animals (shelter and owned) showed no differences in prevalence of *E. multilocularis* (and other parasites detected by PCR and coproscopy) in both cats and dogs. Only results of coproscopic examination of dogs showed that in the shelter there was significantly higher prevalence of all helminths (*χ*^2^ = 9.75, *df* = 1, *P* = 0.0018) and all nematodes (*χ*^2^ = 5.73, *df* = 1, *P* = 0.0166) when analysed together (Table 2).

Among the examined animals there were 42 female and 25 male cats, and 106 female and 162 male dogs. No significant differences in prevalence of parasites between males and females were observed in both cats and dogs. Data concerning age were as follows: 34 cats and 59 dogs were younger (≤ 1 year) and 33 cats and 209 dogs were older (> 1 year). A statistically higher prevalence of tapeworms (combined results of coproscopy and PCR) was observed in older animals (> 1 year) than in younger animals (≤ 1 year): cats: 39.4 *vs* 14.7% (*χ*^2^ = 4.01, *df* = 1, *P* = 0.0451); dogs: 14.8 *vs* 1.7% (*χ*^2^ = 6.35, *df* = 1, *P* = 0.0117). Data concerning anthelminthic treatment history concerned only privately-owned animals. According to owner declarations, 32 cats and 122 dogs were dewormed, and 7 cats and 23 dogs were not treated during the past year. All dewormed animals were treated with drugs recommended for control of both nematodes and tapeworms. A statistically significant difference was observed only in dogs: more dogs were infected by helminths (combined results of coproscopy and PCR concerning all detected helminths) in the untreated group (52.2%) than in the dewormed group (17.2%) (*χ*^2^ = 11.54, *df* = 1, *P* = 0.0007).

The highly endemic status of the investigated area was confirmed by the examination of red foxes. Among 110 examined, 53 were positive for *E. multilocularis* in SCT (48.2%; 95% CI: 39.1–57.4%). The mean intensity was 1966 worms per fox (standard deviation, SD = 7631.5; coefficient of variation CV = 388.1%).

## Discussion

To our knowledge, the present study represents the first time that *E. multilocularis* has been detected in cats in Poland. The previous study that focused on this parasite in cats in Poland [17] showed negative results. These results are probably connected with the origin of the animals. Most of them (37/40) came from Pomorskie Province (NUTS2 PL63) where *E. multilocularis* prevalence in red foxes is low [15]. It is unlikely that *E. multilocularis* will occur in atypical hosts such as cats in areas with very low prevalence in red foxes (principal definitive host) [2, 26]. In the present study, we examined cats and dogs from highly endemic areas where 48.2% of red foxes were *E. multilocularis*-positive. The relatively low percentage in cats observed in our investigation (6.0%) is not surprising compared to the results obtained in other countries. In France, 7% [4] and 9.3% [3] of cat faeces collected in highly endemic areas (prevalence in red foxes of 34–35%) were positive by qPCR for *E. multilocularis*. An earlier study from France [11] showed this infection in 3 of 81 necropsied cats. Umhang et al. [5] found *E. multilocularis* (using PCR) in 3.1% of cat faeces collected in villages in northeastern France (Ardens). One of the oldest cases of *E. multilocularis* in cats in Europe was reported in central Germany by Flesser et al. [13] who microscopically examined the gastrointestinal tract of 162 cats and found 3 (1.8%) of them positive for this tapeworm. An extensive study (more than 10,000 samples) [12] carried out in Germany and in other European countries on samples submitted to private vet clinics showed 0.23% of cats positive for *E. multilocularis*. This lower percentage could be explained by the use of routine flotation as a preliminary stage before PCR; thus, only samples with taeniid eggs could be identified molecularly. In turn, a study conducted in Switzerland with the use of specific ELISA coproantigens showed 0.76% of cat faeces were positive for *E. multilocularis*, most of which were confirmed by PCR or necropsy [27].

The present study confirmed the presence of *E. multilocularis* in dogs in a highly endemic part of Poland at a low percentage (1.5%) similar to that found a few years ago in the region bordering (and slightly overlapping) the present investigation area [10]. In the latter study, *E. multilocularis* was detected for the first time in Poland in 1.4% of examined privately-owned dogs from rural areas [10]. Studies conducted in other European countries also confirmed the relatively low prevalence of this tapeworm in dogs [2], e.g. Slovakia (2.8%) [7], Lithuania (0.8%) [9], eastern France 0.5% [8] and Germany 0.24% [12].

It would seem that due to their rodent hunting behaviour, cats should have a greater chance than dogs of becoming infected with *E. multilocularis*. Nevertheless, the prevalence in cats was similar to that in dogs. This is probably related to the fact that cats are not a typical definitive host of this tapeworm, and development in the intestine is limited, which was confirmed experimentally [1].

The cat and dog faecal samples came from two groups of animals: privately-owned animals and animals from shelter quarantine cages. *Echinococcus multilocularis* was found only in cats from the shelter, while in dogs it was found in both groups. However, no statistically significant differences concerning *E. multilocularis* prevalence between shelter animals and owned animals were found. The possibility of *E. multilocularis* infection in cats and dogs is associated with rural or urban origin. This was confirmed by a study in France [28] where both urban and rural regions were investigated. These results were very close to ours. *Echinococcus multilocularis* was detected in 3.8 *vs* 6.0% of cats and 1.1 *vs* 1.4% of dogs and all *E. multilocularis*-positive samples, as in our study, came from rural areas. This finding is probably connected with the higher possibility of these animals preying on rodents in rural conditions. Our study included cats and dogs from rural areas and from the shelter. Of course, it is not entirely certain whether the animals delivered to the shelter originally lived in the countryside, but it seems that being a stray also predisposed animals to seeking food by hunting rodents.

In the present study, *E. granulosus* (*s.l.*) was not found in cats or dogs, as in the previous study conducted in this region in dogs [10]. This finding was not unusual because in similar studies in Europe, this tapeworm is not detected at all or found very rarely [9, 12]

There was a difference in the species composition of *Taenia* tapeworms between dogs and cats. Namely, *T. taeniaeformis* (which has rodents as the intermediate hosts) was found most frequently in cats. This is probably due to the naturally greater predisposition for preying on rodents among cats. In contrast, most infected dogs were positive for *T. hydatigena*, whose larvae occur in ruminants or pig internal organs. Moreover, most cases of these tapeworms were found in domestic dogs (probably because of better access to post-slaughter waste). However, in dogs captured and sent to the shelter, *T. serialis*, using lagomorphs as hosts, was found most often.

There was a significantly higher prevalence of tapeworms (*Echinococcus*, *Taenia* and *Mesocestoides*) in the group of older cats and dogs. Tapeworm infections involve the necessity of eating an infected intermediate host (by preying or acquiring dead animals) or having access to slaughterhouse waste. The difference between age groups was likely because older and more experienced animals were able to obtain food much more effectively than younger ones. Moreover, most of the detected tapeworm species may survive in intestines and produce eggs for a year or more after infection of the definitive hosts [29–31].

Our study showed a significantly higher percentage of infection with nematodes among dogs in a shelter than privately-owned dogs. This can be explained by the generally worse health condition of stray animals, which increases susceptibility to parasitic infections. The significant impact of the use of anthelmintics on the reduction in parasite prevalence was demonstrated in dogs only when all helminths together were analysed. No impact was found in cats. Studies conducted in dogs a few years earlier in nearby areas [10] also showed some differences in prevalence, but the results were not significant. In other studies, the effectiveness of anthelmintic treatment to control parasites based on a significant decrease in parasite prevalence was also partially confirmed; in France [32] the efficacy of treatment was reported in only one of two examined locations. In contrast, Sager et al. [33] observed the effectiveness of treatment only in relation to hookworms. According to a recent systematic review [34], the only reliable study concerning the effectiveness of anthelmintic treatment in reduction of *E. multilocularis* infection in dogs was conducted in a village of Savoonga (in a hyperendemic area of St. Lawrence Island, Alaska). The success of this ten-year programme was demonstrated by the reduction in prevalence of infection in rodents from 30 to 5% [35].

*Taenia*-like eggs were found in two of four *E. multilocularis*-positive dogs, and since no *Taenia* spp. DNA was detected, it should be assumed that they were eggs of *E. multilocularis*. However, no positive tapeworm eggs were found in any of the *E. multilocularis*-positive cats. Infection in cats, as non-specific, poor definitive hosts of this tapeworm, is less intensive; eggs are shed in lower numbers and in a shorter period in cats than in dogs or foxes [1, 36]. Studies in northeastern France showed *E. multilocularis* infections in cats (by necropsy and qPCR) [5], but no eggs of this tapeworm were found in faeces, and only immature worms were detected in necropsied cats. However, eggs of *E. multilocularis* can be found in faeces not only in experimental conditions [1] but also in naturally infected cats. For example, the first such case in Japan [37] or in studies carried out recently in France [3] detected eggs of this tapeworm in two of four PCR *E. multilocularis*-positive samples.

## Conclusions

To our knowledge, this study shows for the first time the presence of *E. multilocularis* in cats in Poland and confirms the role of dogs in this infection in highly endemic areas. Dogs and cats may be regarded as epidemiologically important components of the *E. multilocularis* life-cycle because of their closer relationship with humans than sylvatic final hosts.

## Data Availability

Data supporting the conclusions of this article are included within the article. The datasets used and/or analysed during the present study are available from the corresponding author upon reasonable request.

## Abbreviations

SCT: sedimentation and counting technique
PCR: polymerase chain reaction
